# Control of Jasmonate Biosynthesis and Senescence by miR319 Targets

**DOI:** 10.1371/journal.pbio.0060230

**Published:** 2008-09-23

**Authors:** Carla Schommer, Javier F Palatnik, Pooja Aggarwal, Aurore Chételat, Pilar Cubas, Edward E Farmer, Utpal Nath, Detlef Weigel

**Affiliations:** 1 Department of Molecular Biology, Max Planck Institute for Developmental Biology, Tübingen, Germany; 2 Instituto de Biología Molecular y Celular de Rosario, Rosario, Argentina; 3 Department of Microbiology and Cell Biology, Indian Institute of Science, Bangalore, India; 4 Gene Expression Laboratory, Plant Molecular Biology, Faculty of Biology and Medicine, University of Lausanne, Lausanne, Switzerland; 5 Departamento de Genética Molecular de Plantas, Centro Nacional de Biotecnología, Consejo Superior de Investigaciones Científicas, Campus Universidad Autónoma de Madrid, Madrid, Spain; Oregon State University, United States of America

## Abstract

Considerable progress has been made in identifying the targets of plant microRNAs, many of which regulate the stability or translation of mRNAs that encode transcription factors involved in development. In most cases, it is unknown, however, which immediate transcriptional targets mediate downstream effects of the microRNA-regulated transcription factors. We identified a new process controlled by the miR319-regulated clade of *TCP* (*TEOSINTE BRANCHED/CYCLOIDEA/PCF*) transcription factor genes. In contrast to other miRNA targets, several of which modulate hormone responses, TCPs control biosynthesis of the hormone jasmonic acid. Furthermore, we demonstrate a previously unrecognized effect of TCPs on leaf senescence, a process in which jasmonic acid has been proposed to be a critical regulator. We propose that miR319-controlled TCP transcription factors coordinate two sequential processes in leaf development: leaf growth, which they negatively regulate, and leaf senescence, which they positively regulate.

## Author Summary

Short, single-stranded RNA molecules called microRNAs (miRNAs) regulate gene expression by negatively controlling both the stability and translation of target messenger RNAs that they recognize through sequence complementarity. In plants, miRNAs mostly regulate other regulators, the DNA-binding transcription factors. We investigated the downstream events regulated by five TCP (TEOSINTE BRANCHED/CYCLOIDEA/PCF) transcription factors that are controlled by the microRNA miR319 in Arabidopsis thaliana. The miR319-regulated TCPs were previously known to be important for limiting the growth of leaves. By applying a combination of genome-wide, biochemical, and genetic studies, we identified new TCP targets that include enzymes responsible for the synthesis of the hormone jasmonic acid. Our analysis of leaf extracts from plants with increased activity of miR319 confirms that altered expression of the biosynthetic genes leads to changed jasmonic acid levels. These plants show also an altered senescence behavior that becomes more normal again when the plants are treated with jasmonate. We propose that the miR319-regulated TCP factors thus coordinate different aspects of leaf development and physiology: growth, which they negatively regulate, and aging, which they positively regulate.

## Abstract 3

A plant microRNA and its targets turn out to regulate both early and late stages of leaf development: early on, they inhibit growth, while later on, they promote the onset of senescence.

## Introduction

In plants, microRNAs (miRNAs) regulate target genes through miRNA-guided cleavage or translational repression of mRNAs that have highly complementary motifs to the regulatory miRNA. Because of the high sequence complementarity that is apparently required in most cases for miRNA target interaction, computational target identification is much more simple and much less ambiguous than in animals [1–3]. Although translational repression may be more widespread than previously thought by those not familiar with the field [4], much of the available evidence suggests that the sequence requirements for regulation by mRNA cleavage and translational repression are very similar [5–7]. In general, the phenotypes of plants in which target genes have been inactivated by knockout mutations closely resemble those in which the corresponding miRNAs are overexpressed. In addition, even closely related miRNAs can have a unique spectrum of target genes, without evidence for cross-regulation at the level of mRNA cleavage or translational repression [8]. One of the few exceptions appears to be an engineered mutation in a microRNA 398 (miR398) target gene that prevents efficient mRNA cleavage but still allows translational repression [9].

Many miRNAs that are conserved throughout flowering plants target transcription factor genes that control various aspects of development (recently reviewed in [10–12]). Several of these in turn modulate the response to hormones, such as the miR159-regulated *GAMYB* (*GIBBERELLIC ACID MYB*) genes [13–15], or the miR160- and miR167-regulated *ARF* (*AUXIN RESPONSE FACTOR*) genes [16–19]. Another set of *ARF* genes is controlled by *TAS3* (*TRANS-ACTING SIRNA LOCUS 3*), which encodes trans-acting small interfering RNAs (siRNAs) [20–23]. Finally, miR393 regulates a group of related auxin receptors that includes the F-box protein TIR1 (TRANSPORT INHIBITOR RESPONSE 1) [24].

While the vast majority of plant miRNAs have been found by large-scale sequencing [25–31], the first described plant miRNA mutant, *jaw*-D, overexpresses an miRNA, miR319a, that had not been previously identified by deep sequencing [32]. In addition, the major targets of miR319a, a series of related *TCP* transcription factor genes, were also the first targets that were identified experimentally, rather than through computational predictions.

The *TCP*s constitute a plant-specific group of transcription factor genes. Although the conserved TCP domain does not share sequence similarity with other characterized DNA-binding domains, it has been predicted to adopt a basic helix-loop-helix (bHLH) structure. Teosinte Branched1 (TB1) from maize, CYCLOIDEA (CYC) from *Antirrhinum*, and the PCNA promoter binding factors (PCF1 and PCF2) from rice are the founding members of the TCP family [33,34]. TB1, CYC, and its close homolog DICHOTOMA (DICH) control various aspects of plant form, and the mutant effects suggest that they negatively regulate growth [35–37]. The PCFs are also implicated in growth control because they bind to promoter motifs that are essential for the expression of the cell cycle regulator PCNA [33]. The *Arabidopsis* genome encodes at least 24 TCPs, which fall into two major groups, classes I and II [34,38]. In contrast to the class II factors including TB1 and CYC/DICH, class I factors such as TCP20 are positive regulators of growth, and it has been suggested that competition on similar DNA binding sites between class I and class II factors is very important in shaping shoot morphology [38,39].

The five miR319-regulated *TCP*s in *Arabidopsis* belong to class II. This group of *TCP* genes is represented in *Antirrhinum* by *CINCINNATA* (*CIN*) [40]. Like *jaw*-D, in which mRNA levels of *TCP2*, *TCP3*, *TCP4*, *TCP10*, and *TCP24* are all strongly reduced, *cin* loss-of-function mutants have highly crinkled leaves [32,40]. A detailed developmental analysis showed that *CIN* is required for the arrest of cell division in the peripheral regions of the leaf. In *cin* mutants, derepressed growth in the periphery leads to a change from the wild-type form with zero leaf curvature to negative leaf curvature, which is expressed as crinkles that cannot be flattened without cutting the leaf [40]. Conversely, reduced leaf size is seen in *Arabidopsis* as well as tomato plants in which miR319 control of *TCP* genes is impaired [32,41]. Finally, experiments with dominant-negative versions have indicated that all class II TCPs, including those that are not regulated by miR319, have similar effects on plant growth [42].

Leaf history starts with the recruitment of founder cells at the flanks of the shoot apical meristem, which develop into leaf primordia (reviewed in [43]). Cell division in the leaf is terminated by a front of mitotic arrest moving from the distal to the proximal part, after which the leaf gains size by cell expansion. The expanded leaf transforms from a metabolic sink into a source for carbon assimilation. The last stage in the life of a leaf is senescence, during which nutrients are coordinately exported to sink tissue, photosynthesis decreases, and chlorophyll is degraded, visible in the change of leaf color from green to yellow. Finally, the cells die [44,45]. The senescence program includes the differential expression of many hundreds of genes [46–49]. Several signaling molecules are involved in leaf senescence, including salicylic acid, and the plant hormones ethylene, cytokinin, and jasmonic acid (JA) [49–52], although the specific mechanisms by which these hormones control senescence are not well understood.

Here, we reveal a new role of miR319-regulated *TCP* genes, which links leaf morphogenesis with other processes, including JA biosynthesis and senescence. We propose that the miR319/TCP regulatory module coordinates and balances different events that are important for leaf development and physiology.

## Results

### Partially Redundant Activity of *TCP* Genes

**Figure 1:**
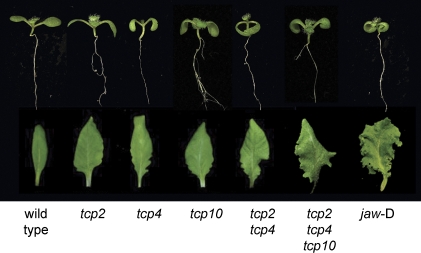
Phenotypes of Plants with Altered *TCP* Activity Ten-day-old seedlings (top) and fully expanded sixth rosette leaves (bottom) of wild-type, various *tcp* mutant combinations, and *jaw*-D plants, in which miR319a, a negative regulator of *TCP2*, *TCP3*, *TCP4*, *TCP10*, and *TCP24*, is overexpressed.

We have previously shown that the *jaw*-D mutant phenotype, with epinastic cotyledons and conspicuously crinkled leaves, is caused by the overexpression of miR319a [32]. To determine the contribution of different miR319 targets to this phenotype, we identified insertional alleles for *TCP2*, *TCP4*, and *TCP10*. Loss-of-function alleles for all three genes had slightly epinastic cotyledons and slightly enlarged leaves (Figure 1). Loss of *TCP4* function in addition caused plants to produce, on average, seven additional leaves before flowering (Figure S1), similar to the delay observed in *jaw*-D mutants [32]. *tcp2 tcp4* double mutants showed a further increase in leaf size, with some signs of crinkling. *tcp2 tcp4 tcp10* triple mutants had the most obvious defects, but were still less strongly affected than *jaw*-D plants were (Figure 1). Interestingly, among plants that overexpressed miR319a from a constitutive 35S promoter, weak lines had bigger, but not crinkly leaves, similar to the *tcp* single knockout plants (Figure S2). In summary, the similar phenotypes of *tcp* loss-of-function mutants and miR319 overexpressers confirmed the conclusion from microarray and other analyses, that the *TCP* genes are the major targets of miR319 [3,8,32]. On the other hand, that all single mutants were only weakly affected indicated partially redundant function of the different *TCP* genes in wild type. These general conclusions are in broad agreement with defects reported for plants expressing dominant negative alleles of *TCP* genes, which mimic many phenotypes of *jaw*-D plants [42].

We also prepared plants that expressed a mutant form of *TCP4* linked to *GREEN FLUORESCENT PROTEIN* (*GFP*) sequences under the control of *TCP4* regulatory sequences (*rTCP4:GFP*). In these plants, *TCP4* mRNA escapes regulation by miR319 due to synonymous changes that reduce sequence complementarity to miR319 [32]. *rTCP4:GFP* plants have a similar, but generally milder phenotype than *rTCP4* plants [32]. Because many more survive to adulthood, we were able to analyze the effects of increased *TCP* levels beyond the seedling stage. Several phenotypic aspects of these plants are opposite to those seen in *tcp* loss-of-function or *jaw*-D mutants. For example, their cotyledons are hyponastic (bent upwards) and hypocotyls are longer than those of wild-type plants (Figure S3A), contrasting with the shorter hypocotyls of *jaw*-D (Figure S3B). The rosette leaves of *rTCP4:GFP* plants were smaller, more rounded, and often darker green than those of wild type (Figure S3C), which contrasts with the larger leaves of *jaw*-D mutants. In summary, these results indicated that a variety of leaf sizes and shapes can be obtained by manipulating the levels of miR319 and its targets, the *TCP* genes, similar to what has been reported for the tomato homologs [41].

### Effects of Altered *TCP* Levels on Genome-Wide Expression Profiles

To identify potential target genes of the miR319-regulated TCPs, we analyzed the results from several microarray experiments (Table S1). We separately compared leaves and shoot apices of wild-type plants with *jaw*-D plants, which have increased miR319a activity and therefore reduced TCP activity. In a third comparison, we analyzed apices from *tcp2 tcp4* double mutants and *rTCP4:GFP* plants, which have increased TCP activity*.* We focused on genes that are likely to be positively regulated by TCPs, as indicated by reduced expression in *jaw*-D or *tcp2 tcp4* plants, or increased expression in *rTCP4:GFP* plants. Because only nine genes were significantly down-regulated in *tcp2 tcp4* double mutant apices, and only two of these were not detected in one of the other three comparisons, we omitted this dataset from further analyses. The weak transcriptional effects seen in *tcp2 tcp4* double mutants are consistent with the weak morphological defects when compared with those of *jaw*-D plants, in which three additional *TCP* genes are strongly suppressed.

**Figure 2:**
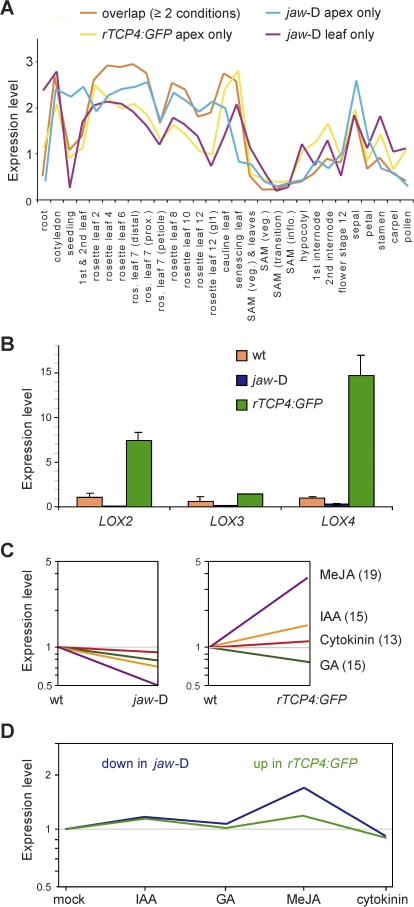
Genes Affected by Altered *TCP* Activity (A) Averaged RNA expression levels of genes affected in different genotypes across a developmental microarray dataset [53]. Shown are genes that are apparently positively regulated by TCPs, because their expression is reduced in *jaw*-D, or increased in *rTCP4:GFP*. See Figure 3A for size and overlap of gene sets. (B) Transcript levels of lipoxygenase genes *LOX2*, *LOX3*, and *LOX4* in apices, measured by qRT-PCR (average of three independent measurements). Error bars indicate standard deviation. (C) Averaged expression levels of hormone biosynthesis genes in shoot apices from different genetic backgrounds, normalized to wild type. Numbers of genes in each pathway given in parentheses. (D) Expression profiles of genes that are changed in *jaw*-D or *rTCP4:GFP*, normalized across five different hormone and control treatments [73].

**Figure 3:**
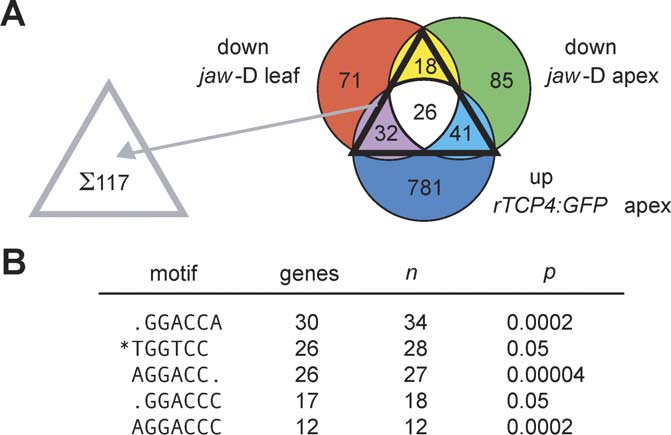
Identification of TCP Target Genes (A) Identification of genes that respond to changes in *TCP* activity in at least two of three comparisons. Selection criteria were a combination of per-gene variance (*p* < 0.05, logit-T, [85]) and common variance (>2-fold). (B) Overrepresented motifs in the promoters of the genes identified in (A). *n* is the number of instances across the genes indicated; *p* is the probability that this is a chance occurrence, corrected for multiple testing. The asterisk indicates a motif that is the reverse complement of the one above.

To obtain first insights into the potential role of the TCP-responsive genes during development, we made use of a developmental microarray dataset [53]. The averaged relative expression levels of the gene sets identified as differentially expressed in each experiment were highly similar, even though there was only partial overlap between them (Figures 2A and 3A). In *rTCP4:GFP* plants, more genes are changed in their expression than in *jaw-*D. One explanation could be that in *rTCP4:GFP* plants, the *TCP4* expression domain is expanded and hence more cells and tissues are affected than in plants with reduced *TCP* activity. In addition, overall *TCP* activity is merely attenuated in *jaw*-D plants, because of the incomplete clearing of *TCP* transcripts by miR319, and because of the partial redundancy between miR319-targeted and nontargeted *TCP* genes, all of which have similar expression patterns (Figure S4) and similar dominant-negative effects [42].

Main sites of expression of *TCP*-responsive genes were leaf-like organs, including cotyledons, rosette leaves, cauline leaves, and sepals, consistent with the tissues affected especially in *jaw*-D plants [32]. That expression of potential *TCP* targets persisted in leaves throughout senescence suggested that the miR319/TCP regulatory module might not only be important early in leaf development, but also during later stages.

### Expression Levels of JA Biosynthetic Genes Affected by miR319/TCP Activity

Using stringent criteria (logit-T per-gene variance *p* < 0.025, common variance > 2-fold), only a single gene, *LIPOXYGENASE2* (*LOX2*), was identified as being affected in the different microarray comparisons (Table S2). *LOX2* was the second most suppressed gene in our original analysis of *jaw*-D plants, after *TCP4* itself [32]. The opposite effects observed in plants with reduced and increased TCP activity, respectively, indicated that TCPs are important determinants of *LOX2* expression levels in the absence of other stimuli known to affect *LOX2* expression, such as wounding [54,55].

*LOX2* encodes a chloroplast-localized lipoxygenase that catalyses the conversion of α-linolenic acid (18:3) into (13S)-hydroperoxyoctadecatrienoic acid, the first dedicated step in the biosynthesis of the oxylipin JA [56]. Apart from LOX2, the *Arabidopsis* genome encodes three other lipoxygenases that are predicted to be chloroplast-localized, LOX3, LOX4, and LOX6 [57]. The expression of *LOX3* and *LOX4* could not be detected by microarray analysis, but more sensitive reverse transcription followed by real-time PCR showed that expression of both genes is reduced in *jaw*-D plants, and increased in *rTCP4* plants as well (Figure 2B).

Since JA is regulated through a positive feedback loop, with JA inducing the expression of its own biosynthetic genes [55,56,58], we examined the effect of miR319/TCP on the entire biosynthesis pathway for JA and other oxylipins, for which 19 genes have been described in *Arabidopsis*. The first steps in JA biosynthesis occur in the chloroplast, and only the JA precursor OPDA (or its coenzyme A [CoA] ester) are transported into the peroxisome, where several rounds of β-oxidation are carried out, leading to the final product, JA [59,60]. We plotted the average expression level of the JA biosynthesis genes against the different genotypes that were subjected to microarray analysis. The average expression of JA biosynthetic genes was approximately 2-fold reduced in *jaw*-D plants compared to wild type, and approximately 4-fold increased in *rTCP4:GFP* plants (Figure 2C). We also analyzed the pathway for the hormones cytokinin, gibberellic acid, and auxin, all of which have been implicated in leaf development or leaf physiology. None of the other three pathways showed as great a contrast between wild-type, *rTCP4:GFP*, and *jaw*-D plants as the JA pathway (Figure 2C, Table S1). When we analyzed publicly available microarray data for JA response, we found the data to be consistent with an effect of miR319-regulated *TCP*s on endogenous JA levels, since several genes that are either down-regulated in *jaw*-D and *tcp2 tcp4* plants or up-regulated in *rTCP4:GFP* plants are induced in wild-type plants treated with methyl jasmonate (MeJA) (Figure 2D). These include genes known from the literature to be responsive to MeJA, such as *PDF1.2* and *COR1* [61,62] (Table S3).

### Identification of a JA Biosynthetic Gene as a TCP Target

To understand how miR319a regulates the JA and oxylipin biosynthesis pathways through the TCP transcription factors, we turned again to the microarray data that we had obtained from the different tissues and genotypes with altered miR319/TCP activity, and we searched for genes that appeared to be positively regulated by TCPs. With slightly relaxed parameters (logit-T per-gene variance *p* < 0.05), we identified a set of 117 genes with consistently changed expression (down *in jaw*-D and up *in rTCP4:GFP*) in at least two of the three analyzed tissues (Figure 3A). In the promoters of this set, the most common motifs were GGACCA and its complement, TGGTCC, which were present at least once in 49 genes (Figure 3B and Table S1).

**Figure 4:**
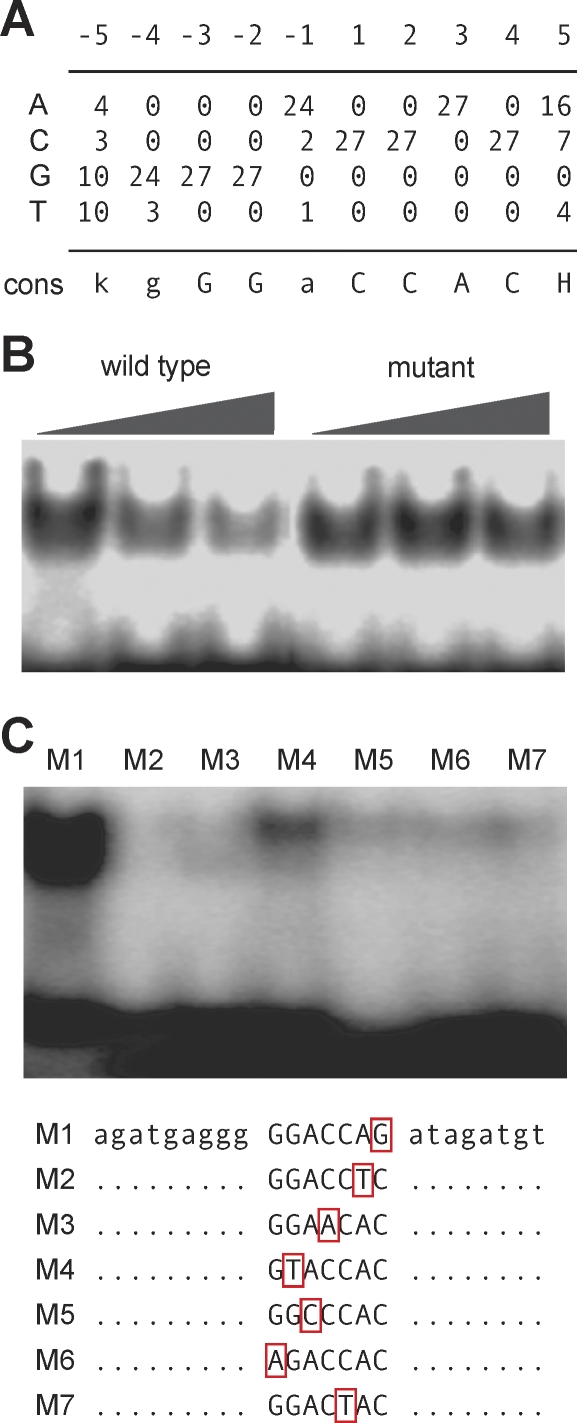
DNA-Binding Specificity of TCP4 (A) Consensus binding motif of TCP4, based on a common sequence found in 25 out of 27 clones selected with recombinant TCP4 protein (see Figure S5). (B) Specificity of TCP4 in vitro binding as shown by EMSAs. Unlabelled double-stranded oligonucleotides that contain the wild-type consensus sequences (agatgggGGACCACatagatgt) or a mutated version (…GGAACAC…) were used in increasing amounts as competitors. (C) TCP4 binding to mutant sites, based on the consensus sequence used in (B).

In parallel, we identified the preferred binding site of TCP4 by in vitro selection [63]. Of 27 clones obtained after ten rounds of selection, 25 contained a variant of the consensus motif gGGaCCAC, which includes as a core the GGACCA motif found in the promoters of TCP-response genes (Figure 4A and Figure S5). Competition experiments with unlabeled oligonucleotides confirmed the specificity of the TCP4 binding site (Figure 4B). Electrophoretic mobility shift assays (EMSAs) with oligonucleotides that contained single base pair mutations indicated some flexibility in the ability of TCP4 to bind its preferred site in vitro (Figure 4C), which may explain why the motif deduced from in silico promoter analysis is only a submotif of the one identified by stringent binding site selection. The complement of the gGGaCCAC motif is related to a sequence, G(T/C)GGNCCC, that is preferentially bound by PCF5, a protein encoded by an miR319-targeted *TCP* gene from rice [38].

In plants, metabolic pathways are often coordinately regulated by the same transcription factors [64], and we found the TCP motif GGACCA in the promoters of eight out of 19 oxylipin biosynthesis genes (Table S2). Only two promoters were expected to have this motif by chance, using the promoters of all *Arabidopsis* genes to determine the background distribution of the GGACCA motif. No such overrepresentation was found in the promoters of 13 auxin, 13 cytokinin, and 15 GA biosynthetic genes, with the GGACCA motif being present in the promoters of only two GA biosynthetic and one auxin biosynthetic genes, and missing in the promoters of cytokinin biosynthesis genes (Table S2).

**Figure 5:**
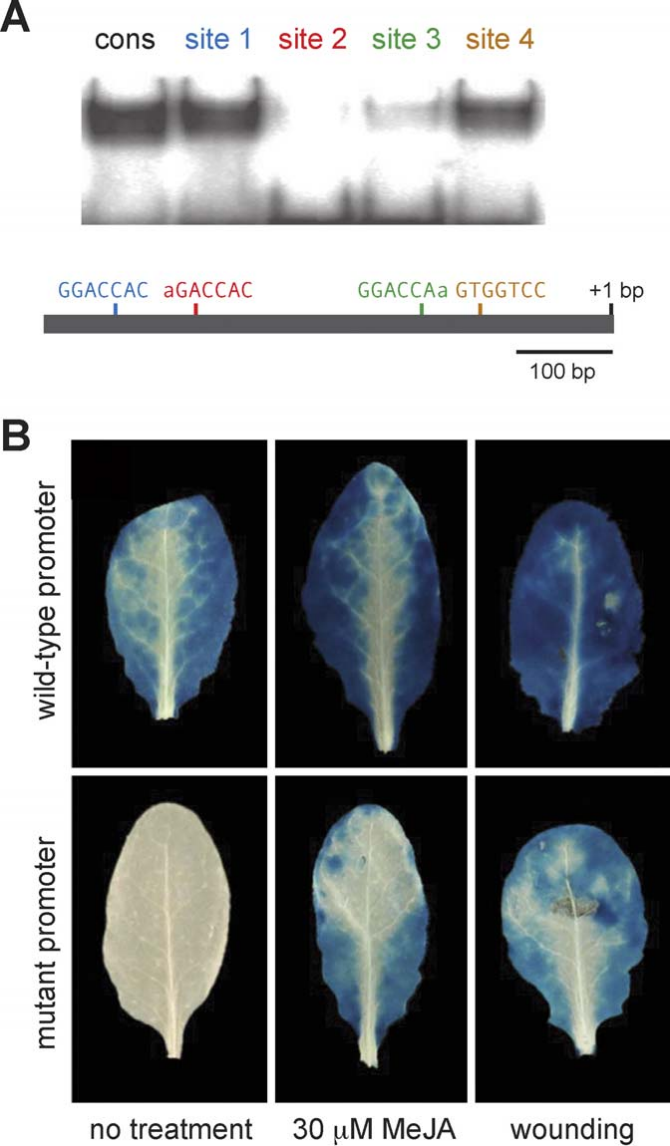
Functional Analysis of TCP4 Binding Sites in the *LOX2* Promoter (A) TCP4 binding to four consensus sites in *LOX2* promoter. (1) tcagatcctGGACCACtgcaataa; (2) tattaattaAGACCACtcgtaact; (3) ttttaagcaGGACCAAaacctaaa; and (4) tagatacaGTGGTCCtcctatgca. consensus motifs are underlined, flanking sequences are from *LOX2* promoter. (*B*) X-gluc (5-bromo-4-chloro-3-indolyl-beta-D-glucuronic acid) assay for β-glucuronidase (GUS) reporter activity. In the mutant promoter, all four TCP4 consensus sites were mutated. MeJA and wounding treatments were for 45 min. The photographs show representative results from 20 independent transgenic lines analyzed for each construct.

To investigate whether the TCP binding sites were indeed required for promoter activity of JA biosynthetic genes, we focused on the *LOX2* promoter, which has four sites with at most one mismatch to the motif GGACCAC. Using double-stranded oligonucleotides covering the potential TCP binding sites in the context of the *LOX2* promoter, we performed EMSAs. The in vitro studies confirmed that TCP4 can bind strongly to at least two of the consensus motifs (Figure 5A). To assess the requirement for these binding sites *in planta*, we constructed two *LOX2:GUS* (β*-glucuronidase*) reporters, one with the wild-type sequence and one in which the four consensus motifs were mutated. In untreated plants, the wild-type reporter had strong GUS activity throughout leaves, similar to what has been reported [55], whereas the mutant reporter had very little activity (Figure 5B). Moreover, the wild-type reporter was less active in *tcp2 tcp4 tcp10* triple mutants (Figure S6), confirming that TCPs positively regulate *LOX2* promoter activity. Together, our findings suggest that the miR319-targeted TCPs directly regulate expression of *LOX2*.

*LOX2* strongly responds to wounding or treatment with MeJA [55,56]. We tested if the mutated *LOX2* reporter lacking TCP binding sites was still responsive to these stimuli. Wounding of rosette leaves or external treatment of plants with MeJA led to strong activation of reporter activity within 45 min (Figure 5B), indicating that TCPs are not involved in these two responses, but rather regulate the developmental aspect of *LOX2* expression.

### JA Levels Affected by Reduced TCP Activity

**Figure 6:**
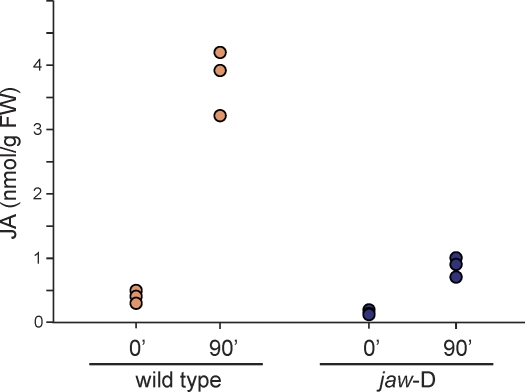
JA Content of Wild-Type and *jaw*-D Plants JA concentration in rosette leaves was measured in triplicate at 0 and 90 min after wounding.

The strongly reduced expression of JA biosynthetic genes in *jaw*-D plants prompted us to ask whether this is accompanied by a reduction in levels of endogenous JA. We measured the concentration of JA *in planta* by using gas chromatography followed by mass spectrometry [65]. JA levels are low in resting tissue of both wild-type and *jaw*-D plants (Figure 6). A difference between wild-type and *jaw*-D plants was most obvious in response to wounding, which strongly induces JA biosynthesis. Near the peak of JA induction in wild type, 90 min after wounding [66], JA levels had substantially increased in both wild-type and *jaw*-D plants, but were about four times lower in *jaw*-D plants. This result is consistent with the observation that wounding can still activate a *LOX2* promoter lacking TCP4 binding sites.

For the microarray analyses, we had used primary *rTCP4:GFP* transformants with relatively strong phenotypes, and found an increase in the expression of JA biosynthetic genes (Figure 2). Unfortunately, only plants with weak, almost wild-type–like phenotypes provided sufficient material for JA measurements, because *rTCP4:GFP* plants with strong phenotypes stay small, have a shorter life span than wild type, and do not produce seeds. No clear differences in JA levels were seen in *rTCP4:GFP* plants with mild leaf phenotypes (unpublished data).

### Senescence in Plants with Altered *TCP* Activity

Neither *allene oxide synthase* (*aos*) mutants, which appear to be completely devoid of jasmonate [66], nor *oxophytodienoate reductase3* (*opr3*) mutants show an obvious defect in an induced senescence assay (Figure S8). On the other hand, it is well known that exogenously applied MeJA can accelerate the final stage of leaf development, senescence (e.g., [67]), and several JA biosynthetic genes, including *LOX2*, are transiently induced during developmental senescence [49]. Thus, JA likely plays a role in the control of senescence, but is not essential for it, as pointed out before [47].

We had noticed that positively regulated TCP targets tend to be expressed at higher levels in older leaves of wild-type plants (Figure 2A). An opposite pattern was seen for genes that were down-regulated in *rTCP4:GFP* plants (Figure S7). Considering that the *rTCP4:GFP* samples analyzed consisted of apices with small, developing leaves, this observations suggested that the developmental age of *rTCP4:GFP* leaves is advanced relative to that of wild-type leaves. The up-regulated genes include several genes encoding *WRKY* transcription factors, so named after the first four amino acids of the conserved motif WRKYGQK, which is the hallmark of this protein family. One of these genes, *WRKY53*, is an important positive regulator of senescence [68,69], which is induced more than 30 times in *rTCP4:GFP* plants, although it lacks TCP4 consensus binding motifs in its promoter (Table S4). The precocious activation in *rTCP4:GFP* of genes that are normally expressed only later during leaf development is consistent with the role of the snapdragon *TCP* gene *CIN* as a regulator of the mitotic arrest front during early stages of leaf growth [40], and suggests a more general role for *TCP*s during leaf aging. This in turn led us to examine the hypothesis that *rTCP4:GFP* plants might show a premature onset of senescence, and that *jaw*-D plants show a delay in senescence.

**Figure 7:**
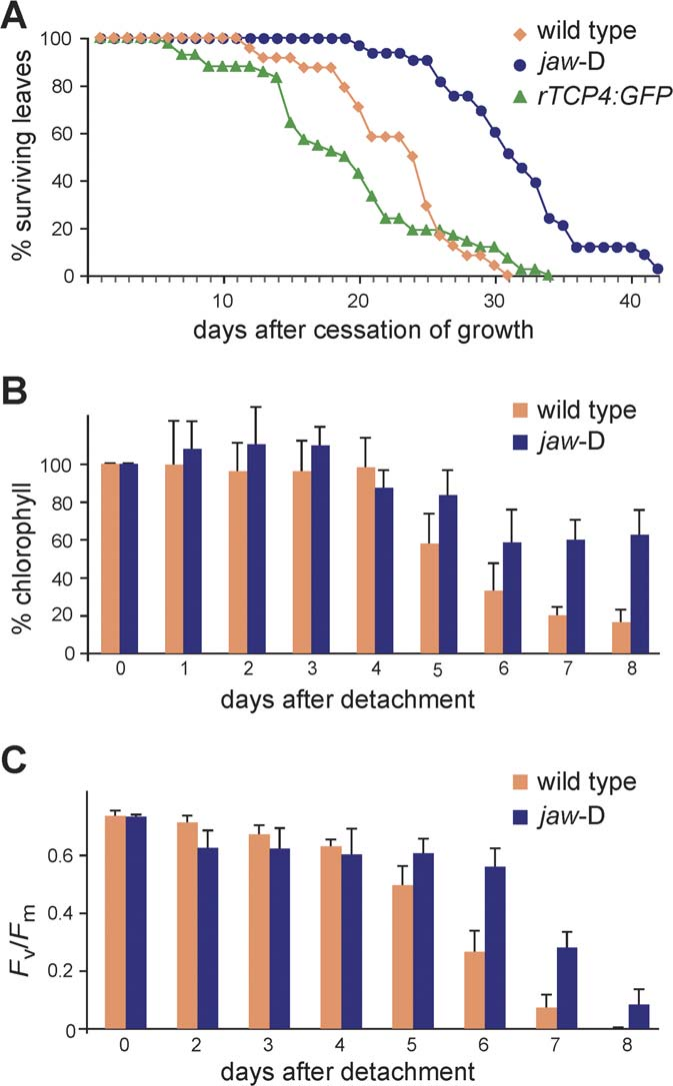
Senescence Responses of Plants with Altered *TCP* Activity (A) Fraction of fifth rosette leaves that still contained chlorophyll after the leaves had stopped growing (wild type, *n* = 28; *jaw*-D, *n* = 31; *rTCP4:GFP*, *n* = 46). (B) Chlorophyll contents in detached leaves of wild-type and *jaw*-D, floated on water in darkness (average of 12 leaves per genotype). (C) Measurements of PSII photochemistry in detached leaves of wild-type and *jaw*-D (average of six leaves per genotype). Error bars indicate standard deviation.

Obvious effects were seen in *jaw*-D plants grown under long days; in these plants, leaf senescence was delayed by about a week (Figure 7A), which is similar to the effects seen in the senescence mutant *oresara9* [70]. In *rTCP4:GFP* plants, senescence was slightly accelerated (Figure 7A), consistent with these *rTCP4:GFP* plants examined having only relatively mild morphological defects.

Incubation of detached leaves in the dark induces senescence within days, and the onset of senescence can be accelerated by treatment with exogenous MeJA [57,67]. Although there are differences between induced and developmental senescence (e.g., [49]), we could confirm the delay observed in on-plant senescence with the in vitro assay. We monitored chlorophyll degradation and maximum efficiency of photosystem II (PSII) photochemistry (*F*_v_/*F*_m_) in detached *jaw*-D leaves incubated in the dark, and found a delayed decline in both these indicators of healthy leaves (Figure 7B and 7C).

**Figure 8:**
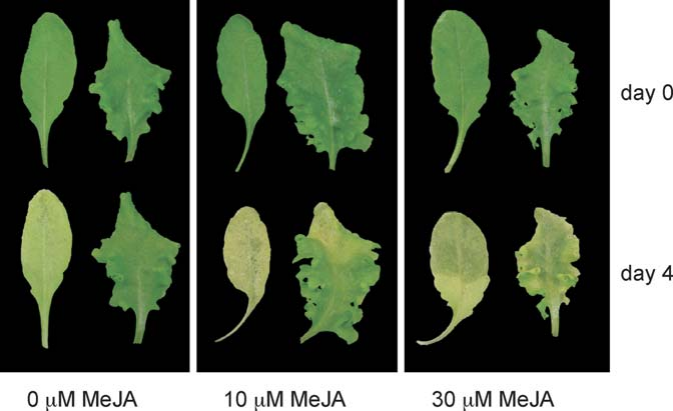
Restoration of Senescence Response in *jaw*-D Detached leaves of wild-type (left leaf of each pair) and *jaw*-D were incubated in darkness with increasing concentrations of MeJA. Note that these assays were carried out to a different time point and in a different laboratory, compared to the experiments shown in Figure 7, which likely explains the differences in senescence progression.

We used the in vitro assay also to determine whether the delayed senescence in *jaw*-D plants is potentially caused by a lack of JA or a defect in JA signaling. When we compared *jaw*-D to wild type, we found that treatment with exogenous MeJA restored the senescence response (Figure 8), consistent with our previous results that TCPs regulate JA biosynthesis, rather than the JA response.

The findings that MeJA was sufficient to restore senescence in *jaw*-D plants, but that JA on its own is apparently not essential for senescence, suggests that JA acts redundantly with other pathways during the control of senescence. One candidate is salicylic acid (SA) signaling, which often antagonizes the effects of JA [71]. However, *jaw*-D plants appeared to be largely normal in their SA response, as deduced from induction of the marker gene *PR1* (Figure S9).

## Discussion

We have investigated the biological roles of miR319 and its targets, a set of five TCP transcription factors, already well known for their effects on leaf growth. We discovered additional functions of the miR319-regulated TCPs in JA biosynthesis and leaf senescence. These findings suggest that TCP transcription factors function throughout leaf development to coordinate the balance between leaf growth, which they negatively regulate, and leaf senescence, which they positively regulate.

### Regulation of Growth and JA Biosynthesis by TCPs

Using a combination of microarray meta-analysis, in vitro DNA binding experiments and reporter gene studies, we identified the *LOX2* gene, which encodes an enzyme catalyzing a key step in JA biosynthesis, as being likely to be directly regulated by TCPs in vivo. The transcriptional response of other genes in the JA biosynthesis pathway and the overrepresentation of a TCP DNA binding motif in this pathway suggest that TCPs directly control additional JA biosynthetic genes. This strategy, coordinated control of metabolic pathways by the same set of transcription factors, is commonly used in plants [64].

Several previous analyses of JA biosynthetic genes, including *LOX2*, have focused on regulatory elements and upstream factors mediating the effects of wounding or MeJA treatment [55,56]. Mutation of the TCP binding sites in the *LOX2* promoter strongly reduced its activity in the absence of stimulation by wounding or MeJA, but it did not abolish the inducibility of the promoter. Our results highlight the importance of developmental control of *LOX2*, and of the fact that developmental regulation can be at least partially uncoupled from transcriptional induction by wounding or JA treatment. This finding is consistent with JA playing not only a role in pathogen and stress response, but also in many developmental processes. Expression of the *TCP* genes themselves is not wound-, pathogen, or MeJA-responsive, as deduced from publicly available microarray data (http://www.weigelworld.org/resources/microarray/AtGenExpress/) [72,73], supporting the conclusion that the *TCP*s represent a pathway of JA regulation that is linked to the developmental program of the plant rather than to environmental responses.

Plants with lower JA levels due to reduced activity of the enzyme encoding genes *DONGLE* (*DGL*) and *OPR3* have been reported to be larger than wild type, while plants that overexpress *DGL* are smaller, similar to plants treated with JA [74,75]. *DGL* shares overlapping activity with a homolog, *DEFECTIVE IN ANTHER DEHISCENCE 1* (*DAD1*), in stamen maturation [76]. *DAD1* in turn is a direct target of the homeotic transcription factor AGAMOUS (AG), which regulates both organ identity during early flower development and organ development during later stages [77]. In light of these related findings, the observation that TCP transcription factors and JA have parallel effects on leaf growth suggests that the oxylipin pathway potentially acts downstream of TCPs in affecting growth. Importantly, several links between JA and cell cycle progression as well as growth have previously been demonstrated (e.g., [78,79]).

### Effects of TCPs and JA on Senescence

Although it is well known that exogenously applied MeJA can accelerate senescence (e.g. [67]), there have been no reports that plants with mutations in the JA biosynthetic pathway are deficient in the senescence program [59,66,76,80–84], which we have confirmed for *aos* and *opr3* mutants using an induced senescence assay (Figure S8). Nevertheless, a bona fide effect of JA on leaf senescence can be deduced from the observation that exogenously applied MeJA fails to induce senescence in the *coronatine insensitive1* (*coi1*) mutant, which is defective in JA signal transduction [57,75,85]. The *coi1* mutant on its own, however, does not show a senescence defect either. Analyses of biosynthetic mutants as well as the *coi1* signaling mutant therefore both suggest that endogenous JA is not limiting for natural senescence.

There is thus an interesting contrast between JA biosynthetic and signaling mutants on the one hand, and *jaw*-D, which has decreased JA levels due to reduced expression of JA biosynthetic genes, on the other hand. We initially considered the possibility that the TCP4 target *LOX2* might be required for the production of additional metabolites that prevent senescence, and that *LOX2* might thereby directly affect chloroplast stability. There are no reports that LOX2 catalyzes processes other than the conversion of α-linolenic acid (18:3) into (13S)-hydroperoxyoctadecatrienoic acid, but the LOX2-catalyzed step can lead to end products other than JA [66]. The functions of these other oxylipins are not well known, and it cannot be ruled out that LOX2-catalyzed products are involved in JA-independent processes that delay senescence. An observation that speaks against such a scenario is that lipoxygenase activity is almost undetectable in leaf extracts of a recently isolated loss-of-function *lox2* mutant. Similar to other JA biosynthetic mutants, these *lox2* mutants do not show obvious changes in their senescence program (L. Dubugnon and E. E. Farmer, unpublished data).

We therefore propose an alternative scenario, namely that miR319-regulated TCPs control leaf senescence by regulating not only JA biosynthesis, but also a second, as-yet unidentified pathway that suppresses senescence in wild-type plants. We speculate that inactivation of the endogenous JA pathway alone is not sufficient to delay precocious senescence, due to such a second, redundantly acting pathway. Because of their parallel effects on senescence, JA alone should be, however, sufficient to induce senescence, both in wild-type plants and in *jaw*-D plants, which presumably lack activity of both pathways. Many genes activated in *rTCP4:GFP* plants are progressively up-regulated during leaf development, including *WRKY53*, an important positive regulator of senescence [68,69], suggesting perhaps a more general role for *TCP*s during leaf aging.

Most conserved plant miRNAs affect transcription factor genes with important roles in development [11,12], but in vivo targets that mediate the effects of these transcription factors are largely unknown. Our identification of targets of miR319-regulated TCPs thus provides an important advance in the understanding of small RNA–controlled regulatory networks. In addition, it demonstrates that the function of miRNA-controlled transcription factors is not limited to the modulation of downstream hormonal responses [13–16,18], but that miRNAs may, in addition, regulate development through effects on hormone biosynthesis.

## Materials and Methods

### Plant material.

Plants were grown at 23 °C. All experiments were done under long days (16 h light), except for microarray analyses, which were with plants grown under short days (8 h light). Regular illumination was 125 μmol m^−2^ s^−1^. For low light conditions, intensity was reduced to 15 μmol m^−2^ s^−1^. Origin of *tcp* mutants and gene identifiers are given in Tables S5 and S6. Wild type was Columbia (Col-0), unless stated otherwise.

### Microarray analyses and promoter motif discovery.

Microarray analyses using the Affymetrix ATH1 platform were performed as described [86]. For the collection of apices (including the youngest leaf primordia), plants were dissected under a stereomicroscope, and all leaves with visible petioles were removed and discarded. Tissue was harvested directly into liquid N_2_. Differentially expressed genes were identified with a combination of per-gene variance (calculated using logit-T [87]) and common variance based on expression estimates using gcRMA (http://www.bioconductor.org), a modification of the robust multi-array analysis (RMA) algorithm [88]. Accession numbers for microarray experiments are GSE518 (*jaw*-D) [32], and E-MEXP-469 (*rTCP4:GFP* and *tcp2 tcp4*). Microarray data for hormone treatment were downloaded from http://www.arabidopsis.org; TAIR accession numbers are 1007965964 (JA), 1007965859 (auxin), and 1007966175 (GA).

Six to eight–nucleotides-long overrepresented motifs were identified using a routine implemented in Genespring GX 7.3.1 (Agilent Technologies, California). Promoters were defined as 800 nucleotides upstream of the initiation codon, and exact matches among positions −800 to −10 were considered. The frequency of each individual motif in the 117 genes that changed in at least two conditions was compared to the frequency of the same motif in promoters of other, randomly chosen genes. The Ath1_02_04 annotation was used.

### Expression analyses.

Real-time RT-PCR using the Opticon Continuous Fluorescence Detection System (BioRad) was performed as described [86]. GUS staining was carried out as described [89].

### Protein expression and purification.

The TCP4 expression construct pRSETC-TCP4-1, designed to express the amino-terminal, 224–amino acid fragment of TCP4 including the DNA-binding domain, was transformed into the Escherichia coli strain BL21 (DE3) pLysSpSBET. 100ml LB containing 100μg/ml ampicillin was inoculated with 1 ml of overnight culture and grown at 37 °C to mid-log phase. Recombinant protein expression was induced with 1 mM isopropyl β-L-thiogalactoside (IPTG). Cells were harvested after 3 h of induction. Cells were lysed by sonication in 2 ml of lysis buffer (50 mM NaH_2_PO_4_, 300 mM NaCl, 10 mM imidazole, 1 mg/ml lysozyme). The lysate was centrifuged and the supernatant was loaded onto a Ni-NTA spin column (Qiagen). Recombinant protein was eluted in 150 μl volume containing 500 mM imidazole. Eluted protein was dialyzed against 50 mM NaH_2_PO_4_, 300 mM NaCl and 10% glycerol for 6 h. Purification was monitored by protein blot using anti-His HRP conjugate antibodies (Qiagen).

### Random binding site selection.

Methods described earlier [63] were used. The double-stranded oligonucleotide targets (R704), which contained random 18-mer sequences flanked by 19 bp defined sequences on both ends, were prepared by annealing oligonucleotides R704 (GGAAACAGCTATGACCATG [N]_18_ ACTGGCCGTCGTTTTAC) and 704 (GTAAAACGACGGCCAGT) followed by primer extension with Klenow fragment. Recombinant protein was incubated with 3.6 μg of double stranded R704 in 15 μl of 1 X binding buffer containing 0.1 M KCl, 10 ng of salmon sperm DNA, and 10 μg of bovine serum albumin (BSA). The DNA-protein complex was separated by polyacrylamide gel electrophoresis, bound oligonucleotides were eluted from the gel and dissolved in 20 μl of TE. The recovered DNA was amplified by 14 cycles of PCR with primers 703 (GGAAACAGCTATGACCATG) and 704. The PCR product (30 μl) was extracted with phenol/chloroform and ether, and 10 μl was subjected to next round of selection. With each round of selection, the number of PCR cycles was reduced by one cycle to avoid generation of high–molecular weight PCR products. The DNA from the tenth round of selection was amplified by PCR, purified on a 20% polyacrylamide gel and cloned into the pGEM-T Easy vector (Promega) for sequencing.

### EMSAs.

Double-stranded DNA probes were generated by annealing oligonucleotides and primer extension with [α-^32^P]-dCTP using Klenow enzyme. The binding reaction was carried out in a total volume of 10 μl containing ∼10 fmol of oligonucleotide probe, 1 X binding buffer (20 mM HEPES-KOH, pH 7.8, 100 mM KCl, 1 mM EDTA, 0.1 % BSA, 10 ng herring sperm DNA, and 10% glycerol) and 5–100 ng of recombinant protein. The mixture was incubated for 30 min at room temperature and loaded on 6% native polyacrylamide gel. Electrophoresis was conducted at 4 V/cm for 45 min in 0.5 x TBE electrophoresis buffer at room temperature. The gels were autoradiographed using a phospho-imager.

### Site-directed mutagenesis.

Mutagenesis was carried out using the QuikChange Multi Site-Directed Mutagenesis Kit (Stratagene), according to the manufacturers instructions. Primer sequences are available on request. The four sites and positions of mutations (with ATG as +1) are: (1) position −1173 to −179, mutated at −1174, −1176; (2) position −944 to −949, mutated at −944, −946, −949; (3) position −432 to −437, mutated at −432, −434; (4) position −300 to −305, mutated at −303, −305.

### Jasmonate quantification.

Protocol 2 of Mueller and colleagues [65] with an oxygen-18 labeled internal standard was used.

## Supporting Information

Figure S1 Flowering Time of *tcp* and *jaw*-D Mutants (87 KB PDF)

Figure S2 Leaf Shape of Weak and Strong miR319a Overexpressors (534 KB PDF)

Figure S3 Hypocotyl and Leaf Phenotypes of *rTCP4:GFP* and *jaw*-D Plants (697 KB PDF)

Figure S4 Expression Profiles of Class II *TCP* Genes in Wild Type (2.46 MB PDF)

Figure S5 Purification and DNA-Binding Properties of TCP4 Protein (1.03 MB PDF)

Figure S6 *TCP* Dependence of *LOX2* Promoter Activity (4.63 MB PDF)

Figure S7 Wild-Type Expression Profiles of Genes Differentially Expressed in *rTCP4:GFP* Plants (103 KB PDF)

Figure S8 Senescence Assay with JA Biosynthetic Mutants (5.87 MB PDF)

Figure S9 Induction of *PR1* after SA Treatment of Wild-Type and Mutant Plants (101 KB PDF)

Table S1 Genes Significantly Changed in *jaw*-D Leaves or Apices, or in *rTCP4:GFP* Plants (126 KB PDF)

Table S2 GGACCA Motifs in Promoters of Hormone Biosynthetic Genes and Changes of Expression Levels in *jaw*-D and *rTCP4:GFP* Plants in Comparison to Wild Type (72 KB PDF)

Table S3 Genes Annotated as Jasmonate Inducible (53 KB PDF)

Table S4 Analysis of Senescence-Associated *WRKY* Genes (53 KB PDF)

Table S5 Origin of *tcp* Mutant Alleles (36 KB PDF)

Table S6 Gene Identifiers (35 KB PDF)

## Abbreviations

*coi1*: *coronatine insensItive1*
*DGL*: *DONGLE*
GA: gibberellic acid
EMSA: electrophoretic mobility shift assay
GFP: GREEN FLUORESCENT PROTEIN
GUS: β-glucuronidase
JA: jasmonic acid
*JAW*: *jagged and wavy*
LOX2: LIPOXYGENASE2
MeJA: methyl jasmonate
miRNA: microRNA
SA: salicylic acid
TCP: TEOSINTE BRANCHED/CYCLOIDEA/PCF
