# Supplementary figures and images for: Control of Jasmonate Biosynthesis and Senescence by miR319 Targets
Source: PLoS Biol. 2008 Sep 23;6(9):e230. doi: 10.1371/journal.pbio.0060230 (PMC2553836; doi:10.1371/journal.pbio.0060230)

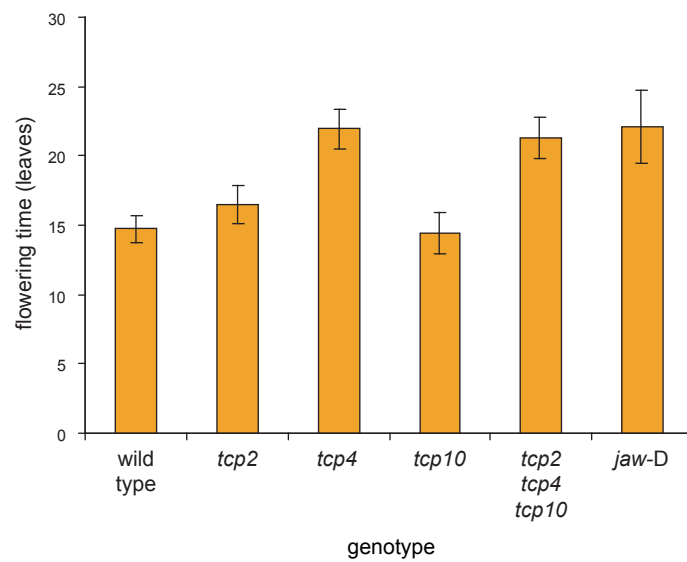

**Figure S1.** Flowering time of different genotypes in long days.

Wild type was Columbia (Col-0).

Supplement: Figure S1 — (87 KB PDF) [file pbio.0060230.sg001.pdf]
