# Supplementary material for: Control of Jasmonate Biosynthesis and Senescence by miR319 Targets
Source: PLoS Biol. 2008 Sep 23;6(9):e230. doi: 10.1371/journal.pbio.0060230 (PMC2553836; doi:10.1371/journal.pbio.0060230)
Supplement: Figure S2 — (534 KB PDF) [file pbio.0060230.sg002.pdf]

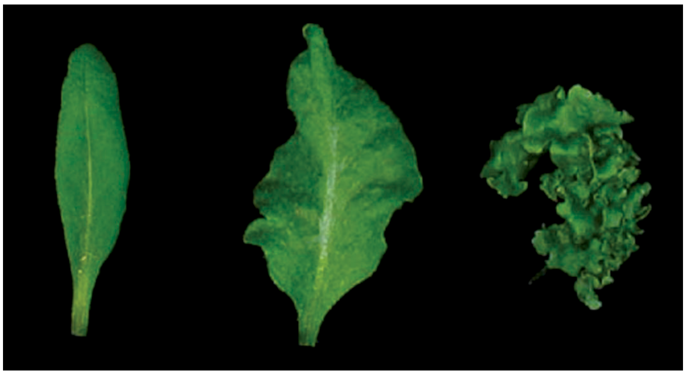

Figure S2. Leaf shape of 35S:*miR319a* plants.

Wild type was Columbia (Col-0). Fully expanded sixth rosette leaves are shown.
