# Supplementary material for: Control of Jasmonate Biosynthesis and Senescence by miR319 Targets
Source: PLoS Biol. 2008 Sep 23;6(9):e230. doi: 10.1371/journal.pbio.0060230 (PMC2553836; doi:10.1371/journal.pbio.0060230)
Supplement: Figure S3 — (697 KB PDF) [file pbio.0060230.sg003.pdf]

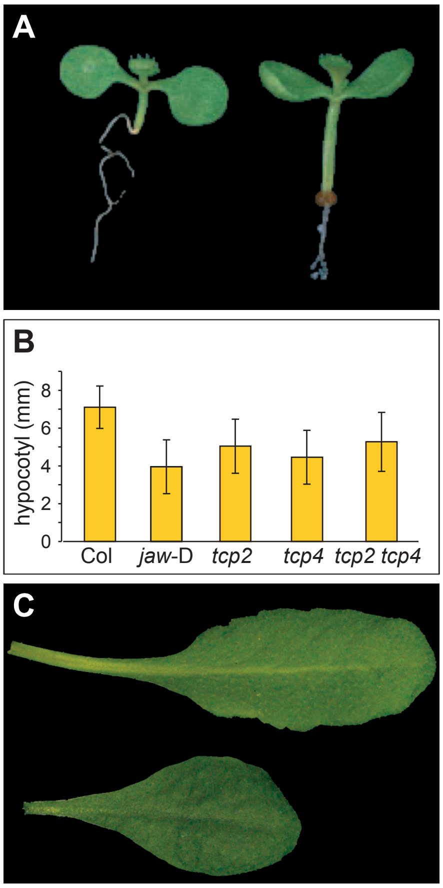

Figure S3. Hypocotyl and leaf phenotypes of *rTCP4:GFP* and *jaw*-D plants.

(*A*) Wild-type (left) and *rTCP4*:*GFP* 10 day-old seedlings. Note elongated hypocotyl in *rTCP4*:*GFP* seedling. (*B*) Hypocotyl length in low light (average of 20 seedlings). (*C*) Sixth rosette leaves of wild type (top) and *rTCP4:GFP*.
