# Supplementary material for: Control of Jasmonate Biosynthesis and Senescence by miR319 Targets
Source: PLoS Biol. 2008 Sep 23;6(9):e230. doi: 10.1371/journal.pbio.0060230 (PMC2553836; doi:10.1371/journal.pbio.0060230)
Supplement: Figure S4 — (2.46 MB PDF) [file pbio.0060230.sg004.pdf]

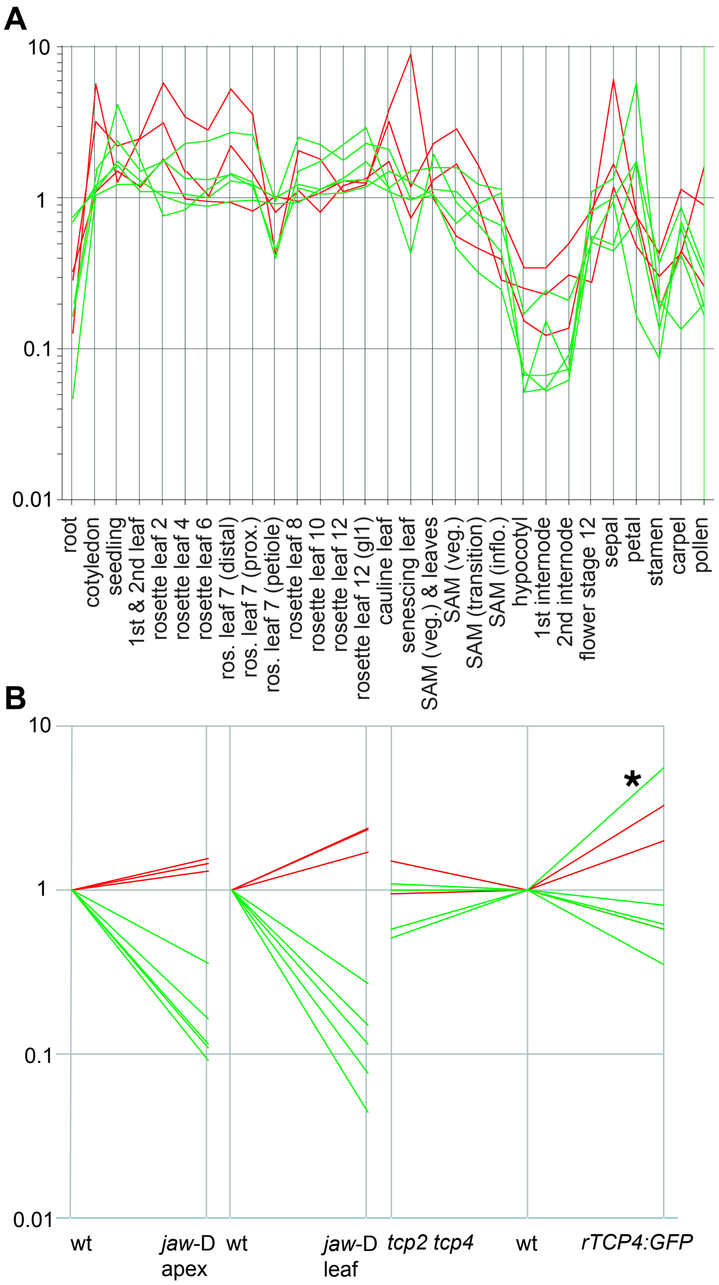

Figure S4. Expression profiles of class II *TCP* genes in wild type.

(A) Expression of five *TCP* genes (*TCP2*, *3, 4*, *10*, *24*) with miR319 target sites (green) and three *TCP* genes (*TCP5*, *13*, *17*) without miR319 target sites (red) across different tissues and stages of *Arabidopsis* development. **(B)** Expression of *TCP* genes (same color code as in A) in different backgrounds. *TCP4* (marked by asterisk) RNA levels are increased in *rTCP4:GFP* plants.
