# Supplementary material for: Control of Jasmonate Biosynthesis and Senescence by miR319 Targets
Source: PLoS Biol. 2008 Sep 23;6(9):e230. doi: 10.1371/journal.pbio.0060230 (PMC2553836; doi:10.1371/journal.pbio.0060230)
Supplement: Figure S5 — (1.03 MB PDF) [file pbio.0060230.sg005.pdf]

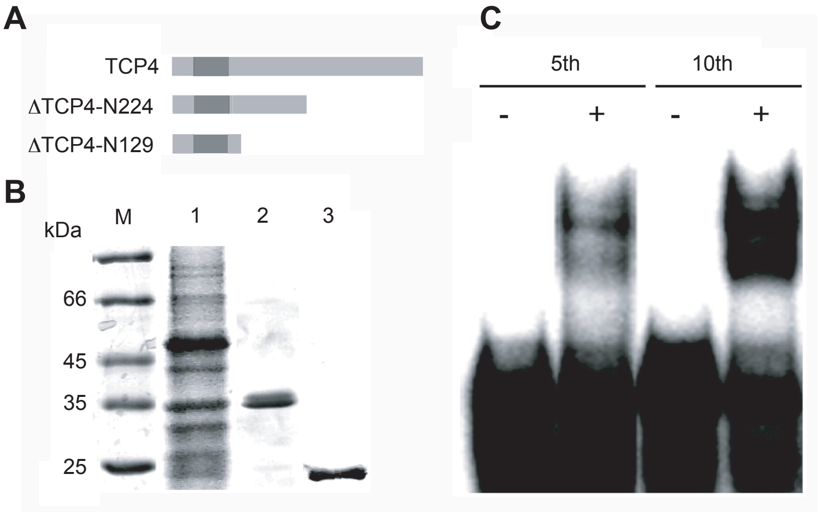

Figure S5. Purification and DNA binding properties of TCP4 protein.

(*A*) Diagrams of full-length and truncated forms of TCP4. The dark-grey box depicts the TCP domain. Numbers indicate the length of the N-terminal fragment (full length TCP4 protein is 420 residues long). (*B*) Polyacrylamide gel showing purification of ΔTCP4-N224 (lane 2) and ΔTCP4-N129 (lane 3) to near homogeneity. Molecular weights of the markers (M) are indicated on the left. The insoluble TCP4 fraction is seen as the thickest band in the pellet fraction after *E. coli* lysis (lane 1). (*C*) Electrophoretic mobility shift assay of the random oligonucleotide pool carried out in presence (+) and absence (-) of ΔTCP4-N224 after 5th and 10th round of enrichment. The high molecular weight bands correspond to the protein-DNA complexes.
