# Supplementary material for: Control of Jasmonate Biosynthesis and Senescence by miR319 Targets
Source: PLoS Biol. 2008 Sep 23;6(9):e230. doi: 10.1371/journal.pbio.0060230 (PMC2553836; doi:10.1371/journal.pbio.0060230)
Supplement: Figure S6 — (4.63 MB PDF) [file pbio.0060230.sg006.pdf]

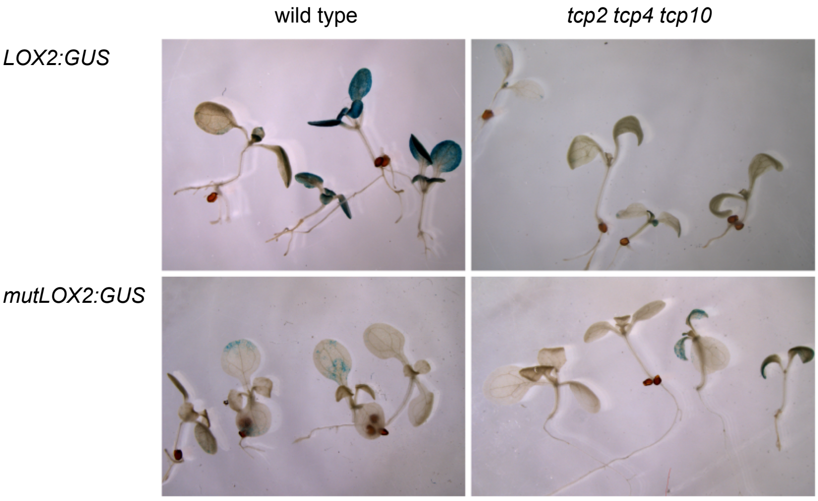

Figure S6. *TCP* dependence of *LOX2* promoter activity.

X-gluc assays for β-glucoronidase (GUS) activity were done with 10 day-old primary transformants. *mutLOX2:GUS* construct contains four mutated TCP consensus binding sites.
