# Supplementary material for: Control of Jasmonate Biosynthesis and Senescence by miR319 Targets
Source: PLoS Biol. 2008 Sep 23;6(9):e230. doi: 10.1371/journal.pbio.0060230 (PMC2553836; doi:10.1371/journal.pbio.0060230)
Supplement: Figure S7 — (103 KB PDF) [file pbio.0060230.sg007.pdf]

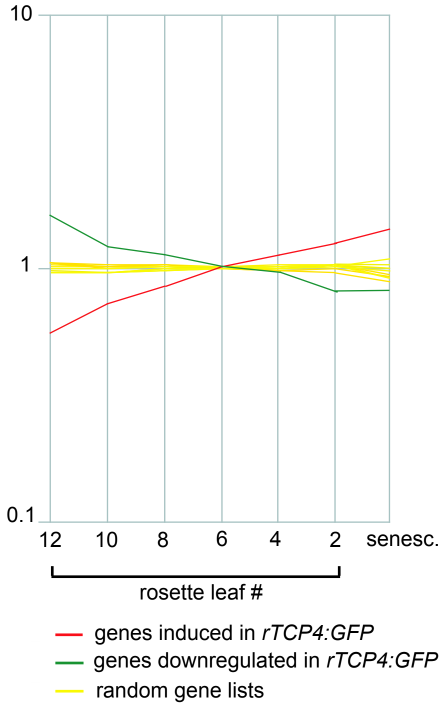

Figure S7. Wild-type expression profiles of genes differentially expressed in *rTCP4:GFP* plants.

Genes were selected based on logit-T per-gene variance p<0.05, common variance >2 fold. Average normalized expression levels in green rosette leaves of wild type, with 12 being the youngest and 2 the oldest, and senescing leaves (senesc.). Ten random gene lists containing 880 genes each were included for comparison. Data are from Schmid M, Davison TS, Henz SR, Pape UJ, Demar M et al. (2005) A gene expression map of *Arabidopsis thaliana* development. Nat Genet 37(5): 501-506.
