# Supplementary material for: Control of Jasmonate Biosynthesis and Senescence by miR319 Targets
Source: PLoS Biol. 2008 Sep 23;6(9):e230. doi: 10.1371/journal.pbio.0060230 (PMC2553836; doi:10.1371/journal.pbio.0060230)
Supplement: Figure S8 — (5.87 MB PDF) [file pbio.0060230.sg008.pdf]

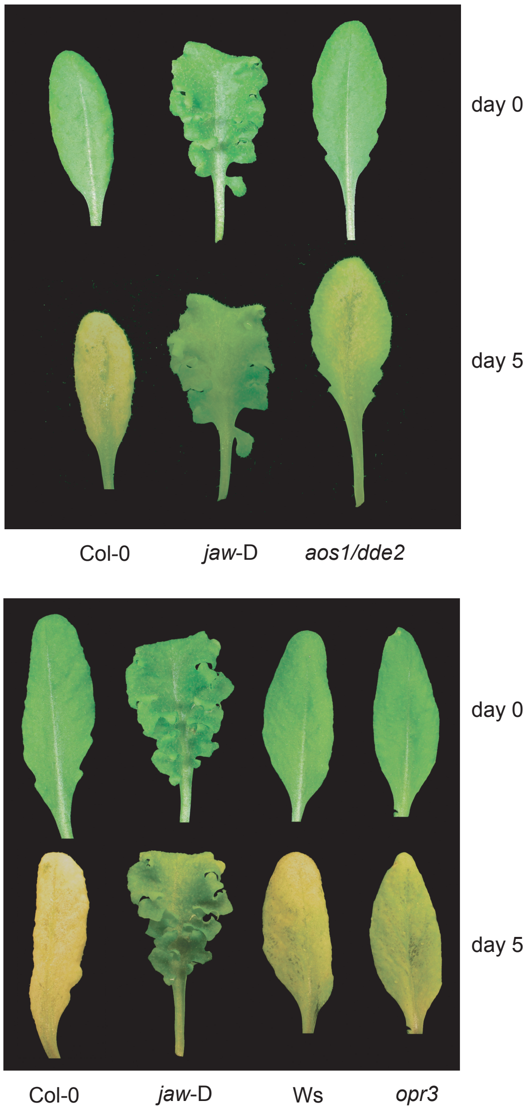

Figure S8. Senescence assay with JA biosynthesis mutants.

Comparison of senescence induced in detached leaves floated on water in the dark. *aos1* (*dde2*) and *jaw*-D are the in Col-0 background, *opr3* in the Ws background.
