# Supplementary material for: Control of Jasmonate Biosynthesis and Senescence by miR319 Targets
Source: PLoS Biol. 2008 Sep 23;6(9):e230. doi: 10.1371/journal.pbio.0060230 (PMC2553836; doi:10.1371/journal.pbio.0060230)
Supplement: Figure S9 — (101 KB PDF) [file pbio.0060230.sg009.pdf]

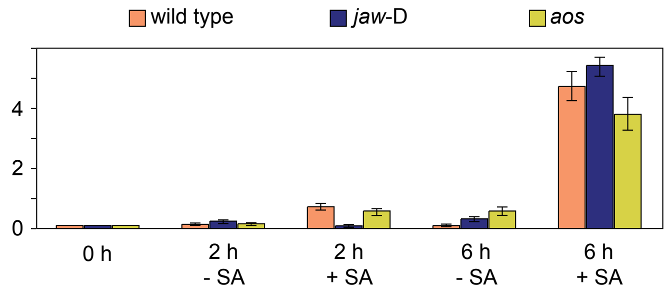


**Figure S9**. Induction of *PR1* after SA treatment of wild-type and mutant plants.

Transcript levels were measured by qRT-PCR. Error bars show standard deviation of three independent measurements.
