# Supplementary material for: Control of Jasmonate Biosynthesis and Senescence by miR319 Targets
Source: PLoS Biol. 2008 Sep 23;6(9):e230. doi: 10.1371/journal.pbio.0060230 (PMC2553836; doi:10.1371/journal.pbio.0060230)
Supplement: Table S1 — (126 KB PDF) [file pbio.0060230.st001.pdf]

Table S1. Genes significantly changing in at least two of three conditions: downregulated in *jaw*-D leaves or apices, upregulated in *rTCP4:GFP* plants (logit-T *p*<0.05 and common variance > 2 fold). Promoters were arbitrarily defined as –800 to –10 bp from ATG.

					Overrepresented motif				
**Gene ID**	**Description**	**fold reduced *jaw*-D apex**	**fold reduced *jaw*-D leaves**	**fold induced *rTCP4:GFP* apex**	**GGACCA**	**TGGTCC**	**AGGACC**	**GGACCC**	**AGGACCC**
At1g03400	2-oxoglutarate-dependent dioxygenase, putative	2.5	2.0	2.1				+	
At1g03850	glutaredoxin family protein	0.7	5.9	15.5		+			
At1g07000	exocyst subunit EXO70 family protein	1.3	2.2	4.3					
At1g07440	tropinone reductase, putative	1.9	1.4	2.6				+	
At1g09070	C2 domain-containing protein	1.4	2.4	4.5					
At1g10340	ankyrin repeat family protein	1.0	4.2	29.8					
At1g13470	expressed protein	2.2	6.8	7.7	+				
At1g19180	expressed protein	4.8	2.2	13.8					
At1g19670	coronatine-responsive protein (CORI1)	3.4	1.3	2.7					
At1g22690	gibberellin-responsive protein, putative	2.0	0.7	3.1					
At1g23130	Bet v I allergen family protein	13.0	2.2	34.3	+				
At1g25560	AP2 domain-containing transcription factor, putative	1.2	2.8	5.3	+				
At1g28600	lipase, putative	4.3	1.7	4.3	+				
At1g30210	TCP family transcription factor, putative	6.1	6.6	0.8					
At1g31680	copper amine oxidase family protein	11.5	8.7	1.7	+		+	+	+

At1g31690	copper amine oxidase, putative	17.4	15.2	2.5					
At1g35710	leucine-rich repeat transmembrane prot. kin., putative	2.7	2.9	16.1					
At1g52800	oxidoreductase, 2OG-Fe(II) oxygenase family protein	1.3	4.1	13.3	+				
At1g53230	TCP family transcription factor 3 (TCP3)	10.8	8.6	0.6					
At1g53885	senescence-associated protein-related	3.0	3.0	1.8					
At1g54040	kelch repeat-containing protein	10.7	5.3	1.6	+		+		
At1g65490	expressed protein	3.8	1.8	17.3					
At1g69870	proton-dep. oligopeptide transport (POT) family protein	2.3	1.6	39.5					
At1g70810	C2 domain-containing protein	2.5	1.3	9.7		+			
At1g80130	expressed protein	1.3	2.3	13.2	+		+		
At2g04160	subtilisin-like protease (AIR3)	7.7	2.3	11.3	+		+		
At2g14560	expressed protein	9.9	22.8	2.3					
At2g17040	no apical meristem (NAM) family protein	1.5	2.9	34.2		+			
At2g19310	expressed protein	2.4	1.2	3.4					
At2g20570	golden2-like transcription factor (GLK1)	2.0	1.3	2.9			+	+	+
At2g25510	expressed protein	2.4	1.3	2.1					
At2g26440	pectinesterase family protein	1.5	4.4	6.1	+		+		
At2g29290	tropinone reductase, putative	9.5	4.8	1.6	+		+		
At2g31070	TCP family transcription factor, putative	2.8	3.7	0.4					
At2g32160	expressed protein	6.1	7.9	3.6					
At2g33530	serine carboxypeptidase S10 family protein	1.9	3.4	3.2	+		+		

At2g33810	squamosa promoter-binding protein-like 3 (SPL3)	10.2	1.5	23.6		+			
At2g37540	short-chain dehydrogen./reductase (SDR) family prot.	2.3	1.7	6.0	+				
At2g39980	transferase family protein	1.6	4.1	4.5		+			
At2g40100	chlorophyll A-B binding protein (LHCB4.3)	3.4	2.5	0.6	+				
At2g40670	two-component responsive regulator (ARR16)	7.2	2.3	4.1		+			
At2g41090	calmodulin-like calcium-binding protein	2.9	2.3	2.3		+			
At2g43590	chitinase, putative	5.7	0.3	136.9				+	
At2g47130	short-chain dehydrogen./reductase (SDR) family prot.	1.2	4.0	5.3					
At3g01960	expressed protein	3.0	3.2	3.5	+		+		
At3g04210	disease resistance protein (TIR-NBS class), putative	1.8	2.3	2.0					
At3g05660	disease resistance family protein	1.1	2.8	10.1	+				
At3g05730	expressed protein	5.7	13.1	10.1					
At3g10720	pectinesterase, putative	1.9	1.4	2.8		+			
At3g11840	U-box domain-containing protein	1.4	2.5	7.6					
At3g11930	universal stress protein (USP) family protein	3.7	0.8	9.4					
At3g14620	cytochrome P450, putative	1.1	2.3	22.1					
At3g15030	TCP family transcription factor, putative	8.6	22.5	5.6					
At3g16660	expressed protein	2.4	1.6	19.9			+	+	+
At3g16670	expressed protein	22.7	10.2	35.6					
At3g19030	expressed protein	2.0	2.5	12.9					
At3g22231	expressed protein	2.7	9.4	1.4					

At3g22240	expressed protein	2.7	4.8	1.5	+				
At3g23030	auxin-responsive protein (IAA2)	0.8	2.7	4.2					
At3g26230	cytochrome P450 family protein	2.5	1.5	5.9					
At3g28220	meprin and TRAF homology domain-containing protein	3.2	0.9	12.3					
At3g28540	AAA-type ATPase family protein	1.0	5.4	26.4					
At3g45140	lipoxygenase (LOX2)	38.6	2.2	4.5	+	+	+		
At3g48720	transferase family protein	2.6	1.6	5.1		+		+	
At3g50260	AP2 domain-containing transcription factor, putative	1.0	3.6	5.2					
At3g50480	broad-spectrum mildew resistance RPW8 family prot.	2.2	2.4	11.9					
At3g51660	macrophage migration inhibitory factor family protein	3.0	5.2	5.0		+			
At3g52340	sucrose-phosphatase 2 (SPP2)	2.0	1.2	10.1		+			
At3g55980	zinc finger (CCCH-type) family protein	0.9	2.5	9.7					
At3g56400	WRKY family transcription factor	1.8	4.8	2.6			+	+	+
At3g60420	expressed protein	7.1	15.4	17.9	+	+	+		
At3g62550	universal stress protein (USP) family protein	2.1	2.1	3.4	+				
At4g14400	ankyrin repeat family protein	12.3	2.6	2.1					
At4g15440	hydroperoxide lyase (HPL1)	4.7	1.8	4.2	+		+	+	+
At4g15660	glutaredoxin family protein	3.9	2.0	14.4			+	+	+
At4g15680	glutaredoxin family protein	3.1	2.1	5.2			+	+	+
At4g15690	glutaredoxin family protein	3.5	3.2	6.1			+	+	+
At4g15700	glutaredoxin family protein	2.6	2.5	4.7			+		
At4g16760	acyl-CoA oxidase (ACX1)	7.9	9.2	1.6					

At4g17810	zinc finger (C2H2 type) family protein	2.2	2.5	1.4					
At4g18390	TCP family transcription factor, putative	9.2	13.0	0.6					
At4g19960	potassium transporter family protein	2.7	1.3	6.6					
At4g22200	potassium channel protein 2 (AKT2) (AKT3)	2.4	1.2	3.0	+	+	+	+	
At4g23290	protein kinase family protein	6.8	3.5	47.4	+	+			
At4g23300	protein kinase family protein	6.2	6.6	126.1	+	+	+	+	+
At4g32280	auxin-responsive AUX/IAA family protein	1.6	6.2	24.8					
At4g37980	mannitol dehydrogenase, putative (ELI3-1)	27.1	65.8	17.9	+	+			
At4g38950	kinesin motor family protein	3.4	3.4	2.1	+				
At4g39800	inositol-3-phosphate synthase isozyme (IPS 1)	2.8	1.8	2.9	+		+	+	+
At5g02760	protein phosphatase 2C family protein	3.9	10.7	33.1		+			
At5g05600	oxidoreductase, 2OG-Fe(II) oxygenase family protein	2.3	1.3	32.0					
At5g07240	calmodulin-binding family protein	2.7	2.8	1.7		+			
At5g08240	expressed protein	0.8	2.6	7.5	+				
At5g10380	zinc finger (C3HC4-type RING finger) family protein	1.2	2.8	89.6			+	+	+
At5g12050	expressed protein	1.1	4.7	11.2		+			
At5g18600	glutaredoxin family protein	8.5	1.6	8.8					
At5g24210	lipase class 3 family protein	3.0	3.1	14.6					
At5g24530	oxidoreductase, 2OG-Fe(II) oxygenase family protein	2.8	3.4	5.9			+		
At5g35490	expressed protein (MRU1)	3.9	1.2	4.6					
At5g36220	cytochrome P450 81D1 (CYP81D1) (CYP91A1)	3.6	1.0	14.2			+		
At5g41410	homeodomain protein (BEL1)	2.7	1.9	6.1					
At5g44260	zinc finger (CCCH-type) family protein	2.2	3.8	9.3					

At5g44420	plant defensin protein, putative (PDF1.2a)	2.6	5.0	12.1					
At5g44810	putative protein	1.7	2.5	3.3					
At5g46230	expressed protein	1.5	2.3	6.4					
At5g47220	ethylene-responsive element-binding factor 2 (ERF2)	2.3	1.5	6.2					
At5g47370	homeobox-leucine zipper protein 2 (HAT2)	0.6	5.2	3.5		+			
At5g50160	ferric reductase-like transmem. component family prot.	2.2	1.1	3.2	+				
At5g51390	expressed protein	2.0	2.7	3.4					
At5g52750	heavy-metal-associated domain-containing protein	1.0	2.2	15.1					
At5g52900	expressed protein	1.4	5.8	2.4		+			
At5g54610	ankyrin repeat family protein	2.5	5.5	2.9		+	+	+	+
At5g57760	expressed protein	10.3	3.3	9.2	+		+		
At5g59670	leucine-rich repeat protein kinase, putative	3.8	3.7	0.8		+			
At5g60900	lectin protein kinase family protein	2.4	3.4	7.2		+			
At5g60910	FRUITFULL (AGL8)	2.5	1.9	65.9					
At5g67370	expressed protein	2.2	1.3	3.9					
