# Supplementary material for: Control of Jasmonate Biosynthesis and Senescence by miR319 Targets
Source: PLoS Biol. 2008 Sep 23;6(9):e230. doi: 10.1371/journal.pbio.0060230 (PMC2553836; doi:10.1371/journal.pbio.0060230)
Supplement: Table S2 — (72 KB PDF) [file pbio.0060230.st002.pdf]

Table S2. GGACCA motifs in promoters (arbitrarily defined as –800 to –10 bp from ATG) of hormone biosynthetic genes and changes of expression levels in *jaw*-D and *rTCP4:GFP* plants in comparison to wild type.

Oxylipin pathway

Gene ID	Description	GGACCA	fold reduced *jaw*-D	fold induced *rTCP4:GFP*
At1g09400	12-oxophytodienoate reductase		1.01	0.97
At1g13280	allene oxide cyclase		1.03	1.13
At1g17420	lipoxygenase 3		1.69	16.83
At1g17990	12-oxophytodienoate reductase	+	0.95	1.16
At1g18020	12-oxophytodienoate reductase	+	n/a	n/a
At1g19640	jasmonate O-methyltransferase	+	2.17	1.46
At1g55020	lipoxygenase 1		0.75	0.40
At1g67560	lipoxygenase 6		0.92	0.79
At1g72520	lipoxygenase 4		1.30	27.31
At1g76680	12-oxophytodienoate reductase		n/a	n/a
At1g76690	12-oxophytodienoate reductase		0.78	2.11
At2g06050	12-oxophytodienoate reductase	+	1.49	3.61
At3g22400	lipgoxygenase		1.35	0.91
At3g25760	allene oxide cyclase	+	10.00	9.71
At3g25770	allene oxide cyclase		n/a	n/a
At3g25780	allene oxide cyclase	+	1.75	16.22
At3g45140	lipoxygenase 2	+	33.33	4.55
At4g15440	HPL1	+	4.76	4.20
At5g42650	allene oxide synthase		3.45	4.62

Auxin pathway

Gene ID	Description	GGACCA	fold reduced *jaw*-D	fold induced *rTCP4:GFP*
At1g04610	YUCCA3		1.04	0.84
At1g51760	indole-3-acetyl-leu hydrolase		n/a	n/a
At2g22330	CYP79B3 monooxygenase		1.28	3.35
At3g02875	indole-3-acetyl-leu hydrolase		0.85	1.49
At3g44300	nitrilase		1.79	1.56
At3g44310	nitrilase 1 (NIT1)		n/a	n/a
At3g44320	nitrilase 3 (NIT3)		1.05	1.25
At4g08790	hydrolase, acting on carbon-nitrogen bonds	+	0.93	0.74
At4g13260	YUCCA2		0.93	2.12
At4g32540	tryptamine monooxygenase (YUCCA1)		0.59	0.59
At4g39950	CYP79B2 monooxygenase		1.32	15.03
At5g15600	nitrilase		1.18	0.68
At5g20960	indole-3-acetaldehyde oxidase		0.76	1.23
At5g22300	nitrilase		1.00	2.98
At5g56660	indole-3-acetyl-leu hydrolase		n/a	n/a

Cytokinin pathway

Gene ID	Description	GGACCA	fold reduced *jaw*-D	fold induced *rTCP4:GFP*
At1g22400	trans-zeatin-O-D-glycosyltransferase		1.79	2.03
At1g67110	cytokinin trans-hydroxylase		0.81	1.05
At1g68460	IPT1		1.11	1.00
At2g27760	tRNA isopentenyltransferase		1.00	0.85
At2g36750	UDP-glycosyltransferase		1.02	0.99
At2g36800	UDP-glycosyltransferase		n/a	n/a
At3g23630	IPT7		1.04	0.99
At3g63110	IPT3		1.59	2.8
At4g24650	IPT4		1.17	0.95
At5g05870	UDP-glycosyltransferase		0.84	1.14
At5g19040	IPT5		1.00	0.92
At5g38450	cytokinin trans-hydroxylase		1.01	1.00
At5g58600	UDP-glycosyltransferase		0.79	0.72

GA pathway

Gene ID	Description	GGACCA	fold reduced *jaw*-D	fold induced *rTCP4:GFP*
At1g15550	gibberellin 3-beta-dioxygenase	+	1.89	1.44
At1g44090	gibberellin 20-oxidase		0.96	0.91
At1g50960	gibberellin 20-oxidase		0.93	0.89
At1g60980	gibberellin 20-oxidase		1.01	1.12
At1g79460	ent-kaurene synthase		0.85	0.34
At1g80330	gibberellin 3-beta-dioxygenase		1.02	0.97
At1g80340	gibberellin 3-beta-dioxygenase		1.16	0.96
At3g46500	gibberellin 20-oxidase		0.93	0.95
At4g02780	ent-copalyl diphosphate synthase		0.99	1.01
At4g21200	gibberellin 20-oxidase		0.91	1.19
At4g21690	gibberellin 3-beta-dioxygenase		0.93	1.07
At4g25420	gibberellin 20-oxidase	+	1.23	0.75
At5g07200	gibberellin 20-oxidase		1.01	0.97
At5g51310	gibberellin 20-oxidase		0.93	0.93
At5g51810	gibberellin 20-oxidase		0.87	0.87
