# Supplementary material for: Control of Jasmonate Biosynthesis and Senescence by miR319 Targets
Source: PLoS Biol. 2008 Sep 23;6(9):e230. doi: 10.1371/journal.pbio.0060230 (PMC2553836; doi:10.1371/journal.pbio.0060230)
Supplement: Table S3 — (53 KB PDF) [file pbio.0060230.st003.pdf]

Table S3. Genes annotated as jasmonate inducible.

**Gene**	**ID**
COR1	At1g19670
senescence associated protein	At1g83885
senescence associated family protein	At2g23810
PDF1.2b	At2g26020
LOX2	At3g45140
HPL1	At4g15440
coronatine-responsive tyrosine-aminotransferase	At4g23600
PDF1.2a	At5g44420
