# Supplementary material for: Control of Jasmonate Biosynthesis and Senescence by miR319 Targets
Source: PLoS Biol. 2008 Sep 23;6(9):e230. doi: 10.1371/journal.pbio.0060230 (PMC2553836; doi:10.1371/journal.pbio.0060230)
Supplement: Table S4 — (53 KB PDF) [file pbio.0060230.st004.pdf]

Table S4. Analysis of senescence related *WRKY* genes.

Change in expression relative to wild type and presence of potential TCP binding sites are given.

WRKY	fold reduced *jaw*-D apex	fold reduced *jaw*-D leaf	fold induced *rTCP4:GFP* apex	TCP binding motifs				
				GGA CCA	TGG TCC	GGA CCC	AGG ACC	AGG ACCC
18	1.05	4.76	10.07	+		+		
54	1.92	10.00	1.14					
53	0.93	1.23	37.84					
58	0.85	2.50	1.2	+	+			

Promoters were arbitrarily defined as –800 to –10 bp from ATG.
