# Supplementary material for: Control of Jasmonate Biosynthesis and Senescence by miR319 Targets
Source: PLoS Biol. 2008 Sep 23;6(9):e230. doi: 10.1371/journal.pbio.0060230 (PMC2553836; doi:10.1371/journal.pbio.0060230)
Supplement: Table S5 — (36 KB PDF) [file pbio.0060230.st005.pdf]

Table S5. Origin of *tcp* mutant alleles.

Line	Background	Origin
tcp2-1	Col-0	SAIL 562-D05
tcp4-1	Col-0	SAIL 1174-02
tcp10-1	Col-0	SALK _027514
