# Supplementary material for: Control of Jasmonate Biosynthesis and Senescence by miR319 Targets
Source: PLoS Biol. 2008 Sep 23;6(9):e230. doi: 10.1371/journal.pbio.0060230 (PMC2553836; doi:10.1371/journal.pbio.0060230)
Supplement: Table S6 — (35 KB PDF) [file pbio.0060230.st006.pdf]

Table S6. Gene identifiers.

Gene	ID
TCP2	At4g18390
TCP3	At1g53230
TCP4	At3g15030
TCP5	At5g60970
TCP10	At2g31070
TCP13	At3g02150
TCP17	At5g08070
TCP24	At1g30210
LOX2	At3g45140
LOX3	At1g17420
LOX4	At1g72520
